# Contrastive learning with transformer for adverse endpoint prediction in patients on DAPT post-coronary stent implantation

**DOI:** 10.3389/fcvm.2024.1460354

**Published:** 2025-01-13

**Authors:** Fang Li, Zenan Sun, Ahmed abdelhameed, Tiehang Duan, Laila Rasmy, Xinyue Hu, Jianping He, Yifang Dang, Jingna Feng, Jianfu Li, Yichen Wang, Tianchen Lyu, Naomi Braun, Si Pham, Michael Gharacholou, DeLisa Fairweather, Degui Zhi, Jiang Bian, Cui Tao

**Affiliations:** ^1^ Department of Artificial Intelligence and Informatics, Mayo Clinic, Jacksonville, FL, United States; ^2^ McWilliams School of Biomedical Informatics, University of Texas Health Science Center at Houston, Houston, TX, United States; ^3^ Division of Hospital Medicine, Perelman School of Medicine, University of Pennsylvania, Philadelphia, PA, United States; ^4^ Health Outcomes and Biomedical Informatics, University of Florida Health, Gainesville, FL, United States; ^5^ Department of Cardiothoracic Surgery, Mayo Clinic, Jacksonville, FL, United States; ^6^ Department of Cardiovascular Medicine, Mayo Clinic, Jacksonville, FL, United States

**Keywords:** dual antiplatelet therapy, contrastive learning, transformer, predictive modeling, adverse endpoint, drug-eluting coronary artery stent implantation, survival analysis

## Abstract

**Background:**

Effective management of dual antiplatelet therapy (DAPT) following drug-eluting stent (DES) implantation is crucial for preventing adverse events. Traditional prognostic tools, such as rule-based methods or Cox regression, despite their widespread use and ease, tend to yield moderate predictive accuracy within predetermined timeframes. This study introduces a new contrastive learning-based approach to enhance prediction efficacy over multiple time intervals.

**Methods:**

We utilized retrospective, real-world data from the OneFlorida + Clinical Research Consortium. Our study focused on two primary endpoints: ischemic and bleeding events, with prediction windows of 1, 2, 3, 6, and 12 months post-DES implantation. Our approach first utilized an auto-encoder to compress patient features into a more manageable, condensed representation. Following this, we integrated a Transformer architecture with multi-head attention mechanisms to focus on and amplify the most salient features, optimizing the representation for better predictive accuracy. Then, we applied contrastive learning to enable the model to further refine its predictive capabilities by maximizing intra-class similarities and distinguishing inter-class differences. Meanwhile, the model was holistically optimized using multiple loss functions, to ensure the predicted results closely align with the ground-truth values from various perspectives. We benchmarked model performance against three cutting-edge deep learning-based survival models, i.e., DeepSurv, DeepHit, and SurvTrace.

**Results:**

The final cohort comprised 19,713 adult patients who underwent DES implantation with more than 1 month of records after coronary stenting. Our approach demonstrated superior predictive performance for both ischemic and bleeding events across prediction windows of 1, 2, 3, 6, and 12 months, with time-dependent concordance (C^td^) index values ranging from 0.88 to 0.80 and 0.82 to 0.77, respectively. It consistently outperformed the baseline models, including DeepSurv, DeepHit, and SurvTrace, with statistically significant improvement in the C^td^-index values for most evaluated scenarios.

**Conclusion:**

The robust performance of our contrastive learning-based model underscores its potential to enhance DAPT management significantly. By delivering precise predictive insights at multiple time points, our method meets the current need for adaptive, personalized therapeutic strategies in cardiology, thereby offering substantial value in improving patient outcomes.

## 1 Introduction

Coronary artery disease (CAD), the leading cause of death globally, affects around 200 million people worldwide and results in around nine million fatalities annually (1). It remains the most common prevalent heart condition in both the United States and worldwide (2, 3). In 2019, CAD was identified as the single largest contributor to global mortality, highlighting its critical impact on population health (4). Patients with CAD can significantly improve their prognosis through early revascularization, primarily percutaneous coronary intervention (PCI), with drug-eluting stent (DES) implantation as its core part. Following PCI, current guidelines recommend patients receive dual antiplatelet therapy (DAPT), a regimen combining aspirin and a P2Y_12_ receptor inhibitor, to reduce risks of myocardial infarction (MI) and stent thrombosis. However, the management of DAPT poses a great challenge as shorter durations may fail to prevent the recurrence of ischemic conditions, whereas prolonged usage can heighten the bleeding risk. Hence, DAPT remains one of the most intensively investigated interventions in cardiovascular medicine (5). The decision-making process regarding the treatment duration requires a thoughtful evaluation of the trade-offs between ischemic and bleeding risks (6).

To support clinical decision-making, the cardiovascular community has developed various risk-predictive scores. Notably, the DAPT score (7, 8) and the PRECISE-DAPT score (9, 10) are prominent tools derived from clinical trials, designed to aid in determining the optimal duration of DAPT. The DAPT score focuses on the benefits of extending DAPT beyond 1 year (12–30 months), whereas the PRECISE-DAPT assesses the risks and benefits of long (12–24 months) vs. short (3–6 months) DAPT durations. Both scores utilize a manageable number of predictors, providing convenience in assisting clinical practice. However, their performance is relatively modest, with c-scores around 0.70 for risk stratifications (7, 9). Additionally, the clinical trial-based derivation source poses some restrictions, applicable only to a fixed time window and a predefined medication regimen. As treatment strategies evolve, there has been a shift towards more personalized and flexible DAPT regimens, such as de-escalation or abbreviation (11, 12), facilitated by the adoption of newer-generation stents and more effective antiplatelet medications. Concurrently, the availability of extensive healthcare data has paved the way for AI-based innovations in risk assessment (13, 14). Numerous studies have utilized machine learning techniques, such as XGBoost and Random Forest, to predict the risks of adverse events following PCI or acute coronary syndrome (ACS) (15–18). However, among these studies, very few focused specifically on DAPT management. To address this gap, we previously developed AI-DAPT (19), an approach using the Light Gradient Boosting Machine (LGBM) classifier (20) to dynamically forecast adverse outcomes post-PCI with various DAPT durations. While this model demonstrated strong performance, it represents an earlier generation of machine learning methods that primarily depend on structured decision trees.

In pursuit of further advancement, our current research has turned to the transformative capabilities of transformer-based models, which are revolutionizing various fields with their superior ability to handle complex patterns and data relationships. Transformer, popularized by their application in natural language processing (NLP) through models like the generative pre-trained transformer (GPT), operates on the principle of self-attention mechanisms that process input data in parallel. This allows for significantly improved efficiency and depth in modeling the temporal dynamics and interactions, which are critical for accurate survival analysis in clinical settings. In this study, we introduce a cutting-edge approach by combining transformer architecture with contrastive learning—a technique that enhances learning efficiency by contrasting pairs of similar and dissimilar data points (21). The Contrastive learning process involves minimizing a contrastive loss function that decreases the distance between similar data points while increasing the distance between dissimilar data points (21). It has been explored in biomedical research recently. Chen et al. proposed a deep multi-view contrastive learning model using multi-omics data for cancer subtype identification (22). Park et al. utilized deep contrastive learning for efficient molecular property prediction (23). Kokilepersaud et al. developed a contrastive learning-based model to classify the biomarkers in optical coherence tomography scans (24). The contrastive element allows the model to focus on critical features that differentiate outcomes (25, 26), making it especially adept at handling the complexities of post-PCI risk assessment. By leveraging the advanced AI framework, we aim to substantially enhance the accuracy, reliability, and flexibility of risk prediction after DES implantation, facilitating effective, personalized DAPT duration management in CAD patients.

## 2 Materials and methods

### 2.1 Data source and study cohort

We utilized the real-world clinical data from the OneFlorida^+^ Clinical Research Consortium, an integral part of the national PCORnet effort (27, 28). The dataset encompasses longitudinal EHRs for approximately 16.8 million individuals in Florida dating back to 2012 and provides a broad spectrum of patient information, encapsulating demographics, diagnoses, medications, procedures, and lab tests, among others. Our study was approved by the University of Florida Institutional Review Board (IRB) under IRB202000875 and the Mayo Clinic IRB under ID24-001183.

**Figure 1:**
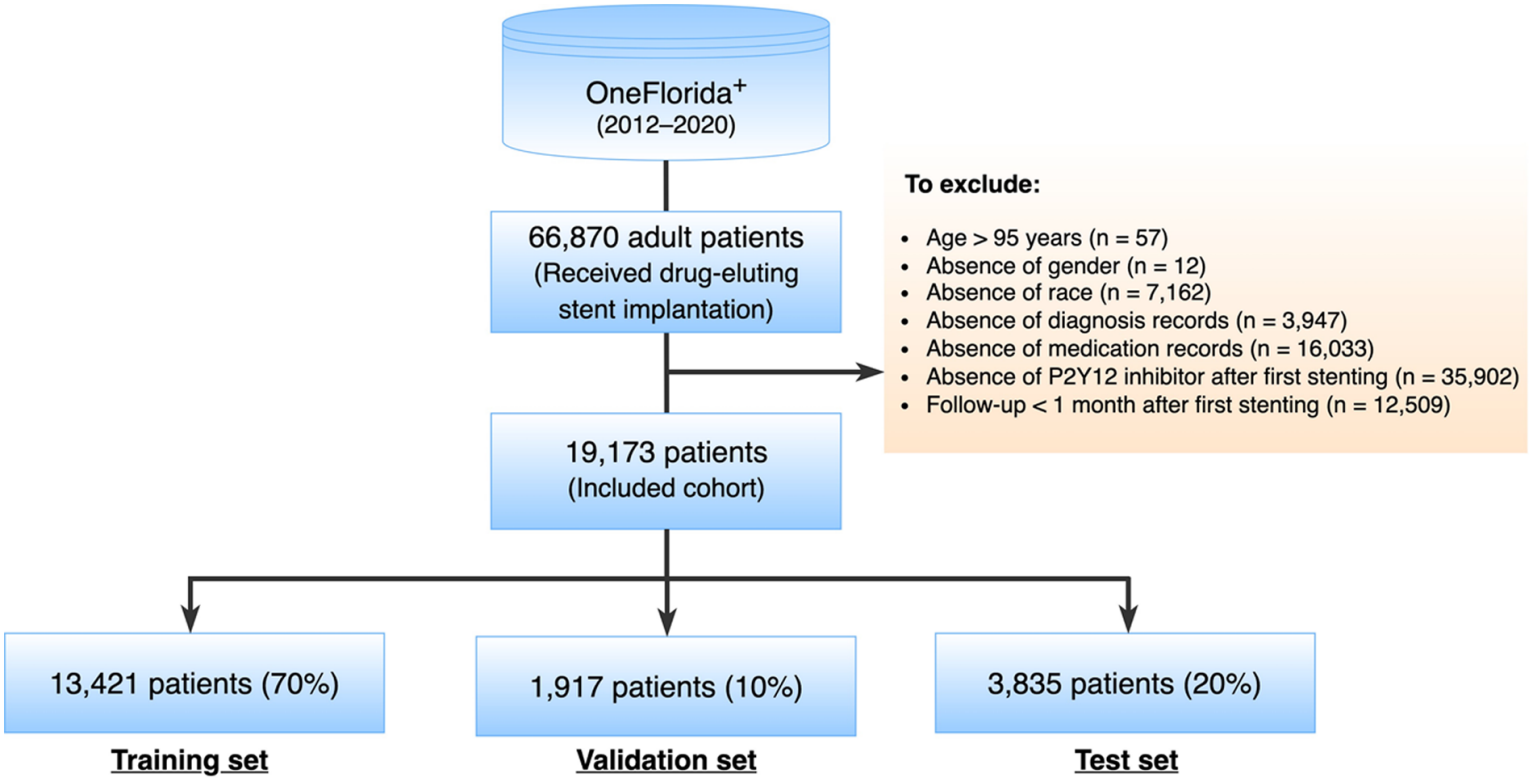
Study cohort selection.

A total of 66,870 adult patients who underwent DES implantation between 2012 and 2020 were identified as potential inclusion for this study. The index date was designated as the date when a patient received the first DES implantation, labeled as time 0. Patients were excluded if they met any of the following criteria: (1) age >95 years at the index date; (2) absence of essential data such as gender or race among their whole records, or missing diagnosis or medication record after the index date; (3) absence of P2Y_12_ inhibitor record after the index date; (4) follow-up less than 1 month after the index date (Figure 1).

### 2.2 Endpoints and characteristics

This study primarily focused on two endpoints: ischemia and bleeding. The primary ischemic endpoint was a composite of acute ischemic heart disease, ischemic stroke, and coronary revascularization. The primary bleeding endpoint consisted of a spectrum from minor to severe spontaneous bleeding as well as blood transfusion. The details of definitions and validation of the phenotyping algorithms could refer to our previous study (19).

Following the approach of the PRECISE-DAPT score, we excluded endpoints occurring during the hospital stay, which were largely related to invasive procedures. Regarding 7 days being the upper limit of current hospitalization trends in patients with ACS, we started event prediction on the 8th day after the initial invasive procedure (the index visit). DAPT exposure was defined as the combinational antiplatelet therapy with aspirin and a P2Y_12_ receptor inhibitor (clopidogrel, prasugrel, or ticagrelor). Due to the over-the-counter availability, aspirin may not necessarily be included as a prescription in the EHR data. Regarding this, we assumed that all patients were on aspirin regardless of whether the aspirin information was captured in their records or not.

This study examined patient characteristics potentially associated with the development of the adverse endpoints, comprising: (1) sociodemographic information, including the age at the index visit, sex, and race/ethnicity; (2) medical history and risk factors, encompassing a range of factors such as prior incidents (bleeding, myocardial infarction [MI], stroke, coronary artery bypass graft surgery [CABG]), lifestyle factors (alcohol abuse, smoking), health conditions (anemia, atrial fibrillation, cancer, chronic kidney disease [CKD], congestive heart failure [CHF], diabetes mellitus, dyslipidemia, hypertension, liver disease, peripheral vascular disease [PVD], and venous thromboembolism [VTE]); (3) concomitant medications, including angiotensin-converting enzyme inhibitors (ACEIs), angiotensin receptor blockers (ARBs), beta-blockers, calcium antagonists, non-steroidal anti-inflammatory drugs (NSAIDs), and statins. The definitions of the comorbidities and generic name sets of the medications were mainly based on the Elixhauser Comorbidity Index, Epocrates Web Drugs, and related studies. The standard terminologies referenced in this study included the International Classification of Diseases, Ninth and Tenth Revision, Clinical Modification (ICD-9-CM, ICD-10-CM) for diagnoses, the ICD procedure coding system (ICD-9-PCS and ICD-10-PCS), Current Procedural Terminology, 4th Edition (CPT-4), and the Healthcare Common Procedure Coding System (HCPCS) for procedures.

### 2.3 Predictive modeling

#### 2.3.1 Contrastive learning with transformer

**Figure 2:**
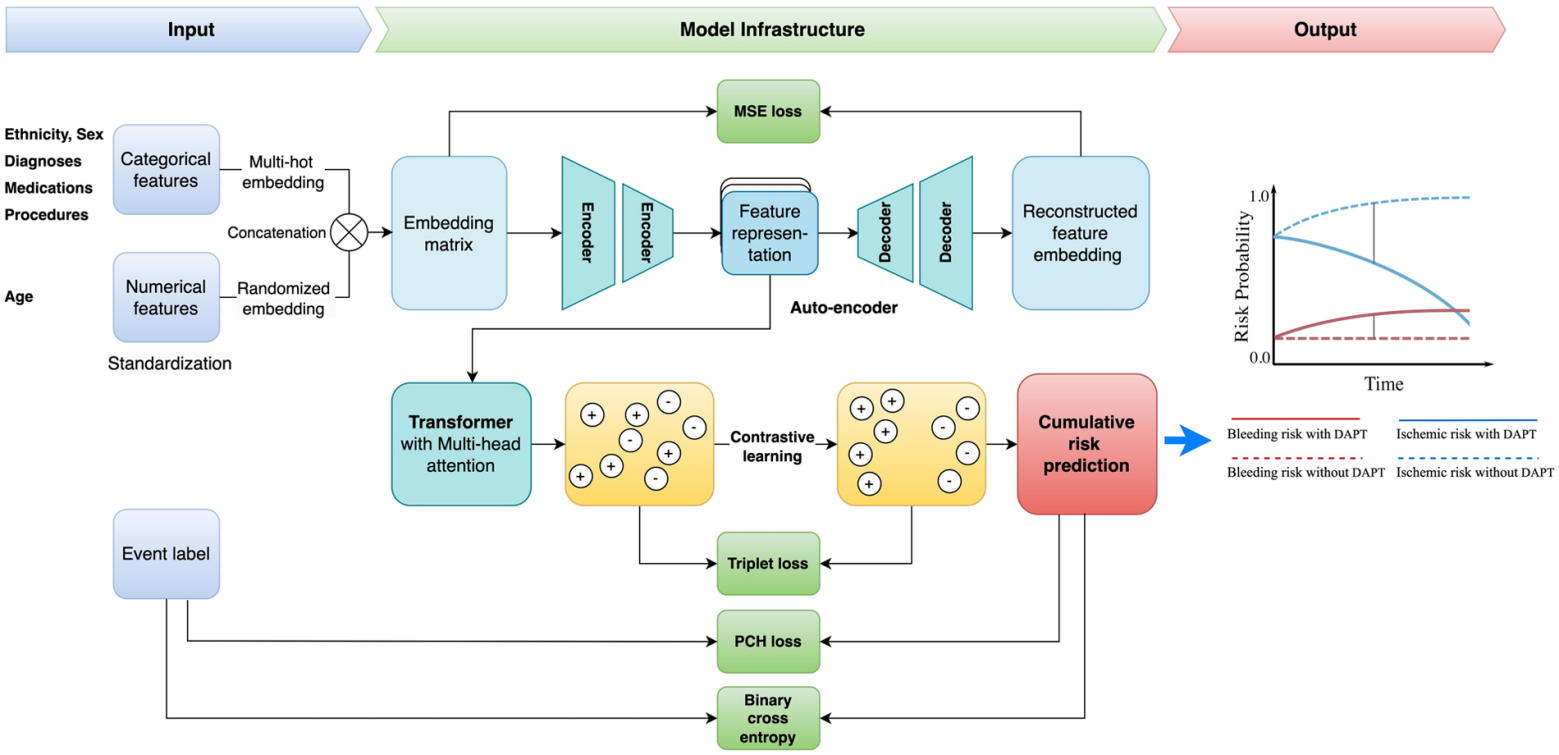
Study framework. DAPT, dual antiplatelet therapy; MSE, mean square error; PCH, piecewise constant hazard.

In this work, we proposed a unified framework for adverse endpoint prediction for patients on DAPT post-PCI. Our approach began with a lower-level auto-encoder (encoder-decoder) model which extracted compressed features from the embedding matrix to reduce dimensionality, which served as input to the multi-head attentional transformer. We performed contrastive learning on the transformer for the improvement of discriminative power and robustness in the parameter space. Specifically, we performed simultaneous optimization for both the event classification and cumulative risk prediction tasks. Given the inherent variability of patient data from different subjects, leverage of the heterogeneous prediction tasks enhances the model's generalization ability towards the noise and variance in the data. The model parameters are optimized with contrastive triplet loss, which seeks to minimize the distance between pairs of samples with the same label and, at the same time maximize the distance between paired samples of different classes. Optimization of this multi-task contrastive target encourages the model to extract meaningful representations that are versatile across the different prediction tasks. Specifically, our model comprises modules of input, autoencoder, transformer encoder, contrastive learning network, and multi-task learning for output. The architecture overview is depicted in Figure 2.

1. **Input Module**. We used the one-hot encoding method to create an embedding lookup table for all codes, then converted values of categorical variables (such as gender and disease) to high-dimensional vectors. We performed standardization transformation for numerical variables (such as age) to achieve a mean of 0 and standard deviation of 1 and then initialized them with a random embedding. As a result, each feature will have an embedding vector with the same size. After that, we concatenated both types of embedding vectors to construct a complete embedding matrix. Each patient's embedding is a 2-dimensional array with an M × N shape, where M is the number of features and N is the embedding size. This embedding matrix was the output of this Input Module.
2. **Autoencoder Module**. Autoencoder is an unsupervised artificial neural network that learns efficient representations of data by compressing input into a lower-dimensional latent space and then reconstructing the original input from this representation. The autoencoder has three parts: an encoder, a bottleneck, and a decoder.
  • Encoder: Learns the hidden features of the input data and then compresses it into a smaller dimension.
  • Bottleneck: Stores the learned representation. It is usually used for further model training and prediction.
  • Decoder: Reconstructs the compressed data to the original dimension. It outputs a synthetic embedding matrix that should be as similar as the input data.

We used mean squared error (MSE) loss ($L_{\mathrm{MSE}}$) to optimize the loss between the input and the reconstructed data:

$$L_{\mathrm{MSE}\;}=\;\frac{1}{N}\sum (h_{i}-h_{i}^{\prime }{)}^{2}$$

Where *N* is the total number of samples, $h_{i}$ is the i^th^ input and $h_{i}^{\prime }$ is the i^th^ reconstructed data. The output of this module is the learned representation from the bottleneck.

3. **Transformer Encoder Module**. Transformer is a popular deep-learning model introduced by Vaswani et al. in 2017 (29). The encoder from the transformer is the pivotal component within the transformer architecture that has emerged as a cornerstone in various machine-learning tasks. It employs an attention mechanism to let the network focus on relevant parts of the input sequence, enhancing its ability to capture long-range dependencies and contextual information. Therefore, the learned representation from the attentive encoder is calculated by:
  $$\mathrm{Attention}\;(Q,K,V)=\mathrm{softmax}\left(\frac{QK^{T}}{\sqrt{d_{k}}}\right)V$$

Where *Q* is the matrix of all query vectors, *K* is the matrix of all key vectors, *V* is the matrix of all value vectors, and $d_{k}$ is the dimension of the key vectors. All query, key, and value matrices are transformed from the input embedding. The $\mathrm{softmax}(\frac{QK^{T}}{\sqrt{d_{k}}})$ calculates the normalized attention scores matrix (or relevant scores matrix) between each feature. The higher attention scores imply greater relevance and force the model to focus on certain parts of the input sequence while generating each output element. As a result, the learned representation is obtained by multiplying the attention score matrix with the value matrix.

4. **Contrastive Learning Module**. Contrastive learning is a self-supervised technique that learns representations by contrasting similar and dissimilar samples. Maximizing agreement between positive pairs (samples from the same class) and minimizing agreement between negative pairs (samples from different classes) enhances the model's ability to discriminate samples from different classes. In this project, we used the triplet loss ($L_{\mathrm{triplet}}$) to minimize the distance between the embedding of positive pairs while maximizing the distance between the embedding of negative pairs. We modified the $L_{\mathrm{triplet}}$ below:
  $$L_{\mathrm{triplet}}=\sum \max(|\left|\;f(x_{i}^{t})-f(x_{i}^{+})|{|}_{2}^{2}+\beta -\right||f(x_{i}^{t})-f(x_{i}^{-})|{|}_{2}^{2,0})$$

Where $f(x_{i}^{t})$ is the embedding of the i^th^ random selected target sample, $f(x_{i}^{+})$ and $f(x_{i}^{-})$ represent the averages of embeddings from either the same or different classes of the target sample, respectively.

5. **Output Module—Cumulative Risk Prediction**. We utilized the shared representation learning network to perform multiple tasks using a shared feature representation. This strategy allows the model to learn new knowledge from different tasks and hence improve model performance. In this project, we designed two distinct tasks: event classification and cumulative risk prediction. The event classification is optimized by the binary cross entropy loss ($L_{\mathrm{BCE}}$) and the risk prediction is updated by the piecewise constant hazard loss function ($L_{\mathrm{pc}}$) (30).
  $$L_{\mathrm{BCE}}=\;-\frac{1}{N}\overset{N}{\underset{i=1}{\sum }}\;y_{i}\log(\;p(y_{i}))+(1-y_{i})\log(1-p(y_{i}))\;\;$$
  $$L_{\mathrm{PC}}=-\frac{1}{N}\overset{N}{\underset{i=1}{\sum }}\;\left(d_{i}\log\lambda_{\kappa (t_{i})}(x_{i})-\lambda_{\kappa (t_{i})}(x_{i})\rho (t_{i})-\overset{\kappa (t_{i})-1}{\underset{\;j=1}{\sum }}\;\lambda_{j}(x_{i})\right)$$

The goal of the piecewise constant hazard loss function $L_{\mathrm{pc}}$ is to evaluate and minimize the discrepancy between the predicted hazard rates (the model's estimate of the risk at any given time) and the actual observed data, including both event occurrences and censored observations. By minimizing this hazard loss, the model learns to predict the risk of events over time while appropriately handling censored data for robust and accurate survival predictions. The loss function consists of three key parts: (1) Maximizing the likelihood of actual events: $d_{i}\log\lambda_{\kappa (t_{i})}(x_{i})$: This term accounts for the likelihood of observing the event at time *t_i_*. If the event is observed (i.e., *d_i_* = 1), this term contributes positively to the likelihood of the predicted hazard rate; (2) Penalizing for survival time predictions: $\lambda_{\kappa (t_{i})}(x_{i})\rho (t_{i})$: This term penalizes the model for incorrect predictions of survival times. The duration of the interval $\rho (t_{i})\;$ scales the predicted hazard rate, reflecting the fact that the event's timing is influenced by how long an individual survives; (3) Penalizing for cumulative risks from earlier intervals: $\overset{\kappa (t_{i})-1}{\underset{\;j=1}{\sum }}\;\lambda_{j}(x_{i})\rho_{j}$: This sum represents the cumulative hazard over previous intervals, accounting for the risk an individual faces in earlier time periods. It helps to adjust for the fact that hazard rates earlier in the study period influence the probability of survival at later times. In this context, *t_i_* is the observed time for the *i-th* subject; *d_i_* is event indicator for the *i-th* subject, specifically, $d_{i}=1$ if the event occurred at time $t_{i}$, $d_{i}=0$ if the data is censored (i.e., the event didn't happen by time $t_{i}$); $\kappa (t_{i})$ is the index of the time interval containing $t_{i}$; $\lambda_{\kappa (t_{i})}(x_{i})$ is the predicted hazard rate for the *i-th* subject in the interval $\kappa (t_{i})$, based on the covariates $x_{i}$ and it represents the instantaneous risk at time $t_{i}$; $\rho (t_{i})$ is duration of the interval that contains $t_{i}$; $\lambda_{j}(x_{i})$ is the predicted hazard rate for earlier intervals (where $j\;<\;\kappa (t_{i})$).

As a result, the final loss function $L_{\mathrm{total}}$ is defined as:

$$L_{\mathrm{total}}=aL_{\mathrm{MSE}}+bL_{\mathrm{BCE}}+cL_{\mathrm{pc}}$$

Where a, b, and c are learned coefficients to balance between each loss function.

#### 2.3.2 Baseline models

We chose three cutting-edge deep learning-based survival models as our baselines: DeepSurv, DeepHit, and SurvTRACE. The LGBM was selected as the backbone model in our previous research (19). Since it is a classification model and does not align with the regression nature of the survival analysis, it was not included as a baseline in this study.

DeepSurv is a modern implementation of the Cox proportional hazards (CPH) model using a deep neural network architecture (31). It uses a multi-layer perceptron architecture to handle survival data, capturing both linear and nonlinear effects from features, and modeling the relationship between covariates and the hazard function. The model takes baseline data as input, processing it through several hidden layers with weights *θ*. Each layer consists of fully connected nodes with nonlinear activation functions, followed by dropout for regularization. The final layer is a single node that performs a linear combination of the hidden features. The network's output is the predicted log-risk function ĥ*θ*(x) (31). A limitation of this model is that it is based on the proportional hazards assumption, which means that the ratio of hazards between individuals is constant over time.

DeepHit is another advanced deep-learning survival model that directly learns the distribution of first-hitting times (32, 33). It comprises a single shared sub-network and a family of cause-specific sub-networks enabling the prediction of a joint distribution of survival times and events. This structure allows it to seamlessly handle multiple competing events using the same features. DeepHit does not assume proportional hazards but it is more complex than DeepSurv and may require more computational resources and data.

SurvTRACE is a state-of-the-art deep learning survival model that leverages a transformer-based architecture to handle complex relationships in the data better to improve model performance (34). Transformer architecture's attention mechanism helps engineer automatic feature learning and increase interpretability. It can handle competing events and multi-task learning through a shared representation network.

#### 2.3.3 Evaluation metrics

We utilized the time-dependent concordance (C^td^) index (34, 35) to measure the performance of cumulative bleeding risk prediction. The traditional concordance index (C-index) evaluates a model's capability to rank the survival times of different individuals accurately. It is determined by the proportion of all possible pairs of individuals for which the model's predictions and the actual outcomes are concordant. The C^td^-index extends this concept by considering survival status at various time points. More specifically, the C^td^-index assesses the model's ability to predict the order of survival times over time by accounting for the censored nature of survival data. In addition to ignoring the bias introduced by the distribution of censoring times, we adapt the inverse probability of censoring weights to obtain a more reliable and robust measure of a model's predictive performance in the presence of censored data. As a result, a meaningful C^td^-index typically ranges from 0.5 to 1.0. A higher value indicates better model performance in ranking survival time or time-to-event outcomes.

We also applied the bootstrapping method to generate a new sampling distribution for model performance evaluation. Specifically, we performed 500 trials to select samples with replacements from the testing set randomly. In each trial, the sample size matched the original testing set size, and the C^td^-index was calculated. The 95% confidence interval (CI) for the C^td^-index was determined using the 2.5th and 97.5th percentiles of the 500 C^td^-index values. To evaluate the significance of the differences between our proposed model and the baselines, we employed Welch's *t*-test (assuming unequal variance) to compare the C^td^-index values derived from the bootstrap samples between our model and baselines.

#### 2.3.4 Model calibration

To assess the predictive reliability of our model, we plotted calibration curves and calculated the Brier scores. A well-calibrated model demonstrates that its predicted probability closely aligns with the actual likelihood of the event happening. The Brier score is a metric commonly used to evaluate the calibration of probabilistic forecasts, by calculating the mean squared difference between the predicted probabilities of an event and the actual outcomes. A lower Brier score indicates better accuracy, with 0 being a perfect score.

### 2.4 Statistical analysis

For descriptive analysis of demographic and clinical characteristics, categorical variables are reported as count (%) and continuous variables as mean (standard deviation, SD). We used the chi-square test for categorical variables and the Kruskal-Wallis test for continuous variables to assess the differences between patients who experienced events and those who did not within 1–24 months. A two-sided *p* ≤ 0.05 was considered statistically significant. Welch's *t*-test was applied to compare model performance, as detailed in the Evaluation metrics section.

## 3 Results

### 3.1 Baseline characteristics

A total of 19,713 adult patients, who underwent DES implantation with more than 1-month records after coronary stenting, were identified as the final cohort from OneFlorida^+^. Of them, 5,088 (26.5%) experienced ischemic events and 3,150 (16.4%) encountered bleeding events within the first year post-DES implantation. The average age of the cohort was 60.4 years with a standard deviation of 11.8. Among them, 12,036 (61.6%) were males.

Regarding the ischemic event, as shown in Table 1, patients with the event were younger than those without the event (59.4 vs. 63.0, *p* < 0.0001). Most clinical characteristics were significantly more prevalent in patients with ischemic events than the non-event group. Notably, 66.4% of patients in the ischemic event group presented with acute ischemic heart disease, compared to only 27.9% in the non-event group (*p* < 0.0001). In terms of medical history, prior conditions like bleeding, ischemic heart disease, and stroke, were more prevalent in the ischemic event group, all showing statistical significance (*p* < 0.05). For comorbidities and risk factors, there was higher prevalence in the ischemic group for conditions like hypertension (92.5% vs. 87.6%), diabetes (62.4% vs. 51.1%), dyslipidemia (93.3% vs. 86.4%), chronic heart failure (CHF) (57.4% vs. 35.6%), anemia (46.1% vs. 29.5%), atrial fibrillation (20.5% vs. 16.2%), smoking (46.4% vs. 34.1%), and alcohol use (10.7% vs. 7.0%) than the non-ischemic group. Interestingly, the prevalence of cancer did not differ significantly (14.4% vs. 14.3%, *p* = 0.83) between the two groups. For medication use, only beta-blockers and statins showed no significant difference while other medications, including the use of ACEI, ARB, calcium antagonists, NSAIDs, and PPIs demonstrated significant differences.

**Table 1:** Baseline characteristics for patients with vs without events (ischemic and bleeding) from 0 to 12 months in the selected cohort of oneFlorida dataset (*N* = 19,713).

Characteristics		Ischemic event			Bleeding event		
		Event (*n* = 5,088, 26.5%)	No event (*n* = 14,085, 73.5%)	*p*-value	Event (*n* = 3,150, 16.4%)	No event (*n* = 16,023, 83.6%)	*p*-value
Age, year, mean (SD)		59.4 (11.9)	63.0 (12.0)	<0.0001*	62.9 (12.5)	61.9 (12.0)	0.0001*
Male, *n* (%)		2,984 (58.6)	9,052 (64.3)	<0.0001*	1,788 (56.8)	10,248 (64.0)	<0.0001*
Ethnicity, *n* (%)	Hispanic	1,159 (22.8)	3,082 (21.9)	<0.0001*	750 (23.8)	3,491 (21.8)	<0.0001*
	NHB	1,050 (20.6)	1,962 (13.9)		594 (18.9)	2,418 (15.1)	
	NHW	2,726 (53.6)	8,185 (58.1)		1,684 (53.5)	9,227 (57.6)	
	Other	153 (3.0)	856 (6.1)		122 (3.9)	887 (5.5)	
Presentation, *n* (%)	Acute ischemic heart disease	3,378 (66.4)	3,932 (27.9)	<0.001*	1,356 (43.0)	5,954 (37.2)	<0.0001*
History, *n* (%)	Prior bleeding	946 (18.6)	1,633 (11.6)	<0.0001*	943 (29.9)	1,636 (10.2)	<0.0001*
	Prior ischemic heart disease	3,734 (73.4)	9,973 (70.8)	<0.0001*	2,512 (79.7)	11,195 (69.9)	<0.0001*
	Prior stroke	919 (18.1)	896 (6.4)	<0.0001*	476 (15.1)	1,339 (8.4)	<0.0001*
	Prior CABG	68 (1.3)	148 (1.1)	0.11	39 (1.2)	177 (1.1)	0.58
Comorbidities and risk factors, *n* (%)	Anemia	2,348 (46.1)	4,151 (29.5)	<0.0001*	2,043 (64.9)	4,456 (27.8)	<0.0001*
	Atrial fibrillation	1,045 (20.5)	2,277 (16.2)	<0.0001*	852 (27.0)	2,470 (15.4)	<0.0001*
	Cancer	735 (14.4)	2,015 (14.3)	0.83	703 (22.3)	2,047 (12.8)	<0.0001*
	CHF	2,920 (57.4)	5,011 (35.6)	<0.0001*	1,863 (59.1)	6,068 (37.9)	<0.0001*
	CKD	1,669 (32.8)	3,207 (22.8)	<0.0001*	1,371 (43.5)	3,505 (21.9)	<0.0001*
	Diabetes	3,175 (62.4)	7,192 (51.1)	<0.0001*	2,031 (64.5)	8,336 (52.0)	<0.0001*
	Dyslipidemia	4,749 (93.3)	12,176 (86.4)	<0.0001*	2,913 (92.5)	14,012 (87.4)	<0.0001*
	Hypertension	4,708 (92.5)	12,334 (87.6)	<0.0001*	2,920 (92.7)	14,122 (88.1)	<0.0001*
	Liver disease	910 (17.9)	1,276 (9.1)	<0.0001*	621 (19.7)	1,565 (9.8)	<0.0001*
	PVD	2,407 (47.3)	4,324 (30.7)	<0.0001*	1,644 (52.2)	5,087 (31.7)	<0.0001*
	VTE	416 (8.2)	463 (3.3)	<0.0001*	283 (9.0)	596 (3.7)	<0.0001*
	Alcohol abuse	546 (10.7)	982 (7.0)	<0.0001*	357 (11.3)	1,171 (7.3)	<0.0001*
	Smoking	2,361 (46.4)	4,808 (34.1)	<0.0001*	1,462 (46.4)	5,707 (35.6)	<0.0001*
Medications, *n* (%)	ACEIs use	1,926 (37.9)	5,703 (40.5)	<0.0001*	1,253 (39.8)	6,376 (39.8)	1.00
	ARBs use	783 (15.4)	1,966 (14)	0.01*	548 (17.4)	2,201 (13.7)	<0.0001*
	Beta-blockers use	2,698 (53.0)	7,551 (53.6)	0.48	1,808 (57.4)	8,441 (52.7)	<0.0001*
	Calcium antagonists use	1,326 (26.1)	4,146 (29.4)	<0.0001*	1,057 (33.6)	4,415 (27.6)	<0.0001*
	NSAIDs use	1,000 (19.7)	2,111 (15)	<0.0001*	682 (21.7)	2,429 (15.2)	<0.0001*
	PPIs use	1,545 (30.4)	6,066 (43.1)	<0.0001*	1,260 (40.0)	6,351 (39.6)	0.72
	Statins use	2,518 (49.5)	7,156 (50.8)	0.11	1,656 (52.6)	8,018 (50.0)	0.01*

ACEIs, angiotensin-converting enzyme inhibitors; ARBs, angiotensin receptor blockers; CABG, coronary bypass artery graft surgery; CHF, congestive heart failure; CKD, chronic kidney disease; NSAIDs, non-steroidal anti-inflammatory drugs; PPIs, proton pump inhibitors; PVD, peripheral vascular disease; VTE, venous thromboembolism.

*p*-values were estimated by the chi-square test for categorical variables and the Kruskal-Wallis test for continuous variables.

* *p* ≤ 0.05 is regarded as significant.

Regarding bleeding events, patients who experienced a bleeding event were older than those who did not (62.9 vs. 61.9, *p* = 0.0001). Similar to the ischemic event findings, most diagnostic characteristics were more prevalent in patients with bleeding events, except for prior CABG, which showed no significant difference. For medication use in the context of bleeding events, except for ACEIs and PPIs, which showed no significant difference between the event and non-event groups, there is a significantly higher usage of ACEIs, beta-blockers, calcium antagonists, NSAID, and statin drugs in the event group vs. non-event group.

### 3.2 Model performance

We assessed the performance of our proposed model against several baselines using the C^td^-index at multiple time intervals post-PCI, specifically in 1, 2, 3, 6, and 12 months.

For the ischemic event prediction, our model consistently outperformed the baseline models (DeepSurv, DeepHit, and SurvTRACE) (as shown in Table 2). Initially, in the first month, the C^td^-index for our model was 0.88, higher than DeepSurv (0.84), DeepHit (0.87), and SurvTRACE (0.87). This trend continued with C^td^-index scores in the second month (0.85), third month (0.84), sixth month (0.83), and 12th month (0.80), consistently surpassing the corresponding baseline values. Notably, the performance advantage was more pronounced in shorter prediction windows.

**Table 2:** Model performance (with 95% CI) across different prediction intervals for the ischemic event^a^.

Models	Prediction windows									
	1-month		2-month		3-month		6-month		12-month	
	C^td^-index	*p*-value	C^td^-index	*p*-value	C^td^-index	*p*-value	C^td^-index	*p*-value	C^td^-index	*p*-value
DeepSurv	0.84 (0.83, 0.86)	<0.001*	0.83 (0.82, 0.84)	<0.01*	0.82 (0.81, 0.84)	<0.01*	0.82 (0.81, 0.83)	<0.05*	0.80 (0.79, 0.81)	0.78
DeepHit	0.87 (0.86, 0.88)	<0.05*	0.84 (0.83, 0.85)	<0.05*	0.82 (0.81, 0.84)	<0.01*	0.79 (0.78, 0.80)	<0.01*	0.77 (0.75, 0.78)	<0.01*
SurvTrace	0.87 (0.86, 0.88)	<0.05*	0.84 (0.83, 0.85)	<0.05*	0.83 (0.82, 0.84)	<0.05*	0.81 (0.80, 0.83)	<0.01*	0.79 (0.78, 0.80)	<0.05*
Ours	0.88 (0.87, 0.89)	Reference	0.85 (0.84, 0.86)	Reference	0.84 (0.83, 0.85)	Reference	0.83 (0.81, 0.84)	Reference	0.80 (0.79, 0.81)	Reference

C^td^-index, time-dependent concordance index.

^a^ The model performance (in C^td^-index) was calculated by employing bootstrapping on the test dataset, and statistical significance (*p*-values) was determined using Welch's *t*-test (assuming unequal variance).

* *p* ≤ 0.05 is regarded as significant.

For the bleeding event prediction, similarly, our model demonstrated superior performance (as shown in Table 3). In 1 month, our model achieved a C^td^-index of 0.82, surpassing DeepSurv (0.79), DeepHit (0.81), and SurvTRACE (0.80). This superiority persisted through the 2nd-month (0.81), 3rd-month (0.82), 6th-month (0.82), and 12th-month (0.77) evaluations, generally outperforming all baselines. For both ischemic and bleeding event predictions, the superiority of our model was statistically significant with most *p*-values less than 0.001.

**Table 3:** Model performance (with 95% CI) across different prediction intervals for the bleeding event^a^.

Models	Prediction windows									
	1-month		2-month		3-month		6-month		12-month	
	C^td^-index	*p*-value	C^td^-index	*p*-value	C^td^-index	*p*-value	C^td^-index	*p*-value	C^td^-index	*p*-value
DeepSurv	0.79 (0.75, 0.82)	<0.01*	0.78 (0.76, 0.81)	<0.01*	0.79 (0.77, 0.82)	<0.01*	0.80 (0.78, 0.81)	<0.01*	0.76 (0.74, 0.78)	<0.05*
DeepHit	0.81 (0.78, 0.84)	<0.05*	0.81 (0.80, 0.83)	0.12	0.81 (0.79, 0.83)	<0.05*	0.81 (0.79, 0.82)	<0.05*	0.76 (0.74, 0.78)	<0.05*
SurvTrace	0.80 (0.76, 0.84)	<0.01*	0.80 (0.77, 0.82)	<0.05*	0.80 (0.77, 0.82)	<0.01*	0.80 (0.78, 0.82)	<0.01*	0.77 (0.75, 0.78)	0.36
Ours	0.82 (0.79, 0.85)	Reference	0.81 (0.79, 0.84)	Reference	0.82 (0.79, 0.84)	Reference	0.82 (0.80, 0.83)	Reference	0.77 (0.75, 0.79)	Reference

C^td^-index, time-dependent concordance index.

^a^ The model performance (in C^td^-index) was calculated by employing bootstrapping on the test dataset, and statistical significance (*p*-values) was determined using Welch's *t*-test (assuming unequal variance).

* *p* ≤ 0.05 is regarded as significant.

The weights assigned to the loss function are as follows: For ischemic event prediction, the weights are 0.3 for $L_{\mathrm{MSE}}$, 0.14 for $L_{\mathrm{BCE}}$, and 0.95 for $L_{\mathrm{PC}}$; For bleeding event prediction, the weights are 0.9 for $L_{\mathrm{MSE}}$, 0.14 for $L_{\mathrm{BCE}}$, and 0.89 for $L_{\mathrm{PC}}$. More hyper-parameters of the model are detailed in Supplementary Table S1.

### 3.3 Calibration curves

**Figure 3:**
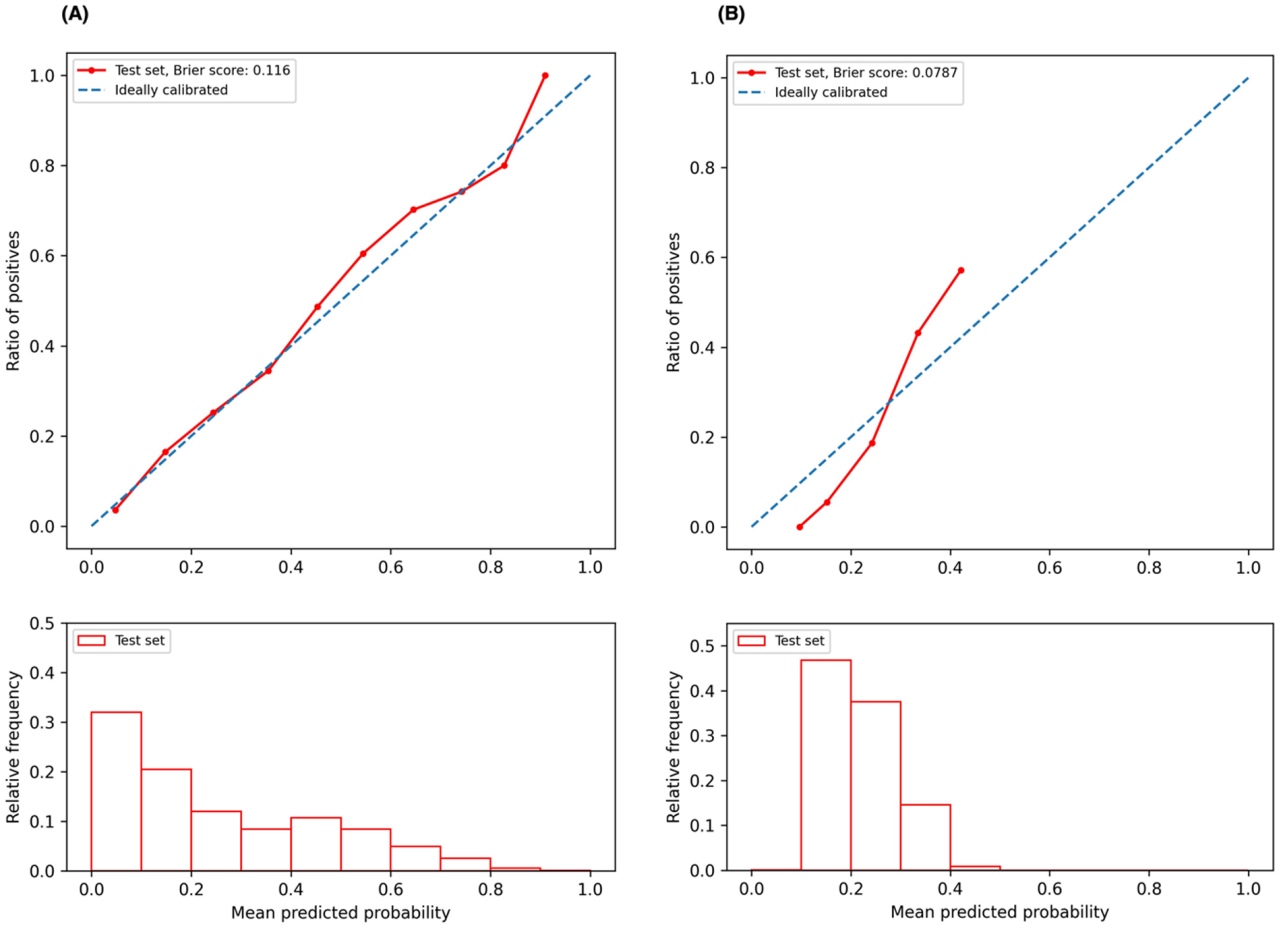
Calibration curves. **(A)** Calibration curve for ischemic event prediction at the end of 12 months; **(B)** calibration curve for bleeding event prediction at the end of 12 months.

We plotted the model calibration curve to assess how well the predicted probabilities of the endpoints align with the actual observed frequencies at the end of 12 months. In Figure 3, the line chart (top) shows the ratio of actual positives in each interval of predicted probabilities, while the histogram (bottom) depicts the relative frequency of predicted probabilities. Figure 3A demonstrates that the predicted probability of ischemic events accurately reflects the actual likelihood, as the predicted probability (red solid line) closely follows the actual likelihood (blue dashed line). The predicted probability for ischemic events ranges from 0 to 1, with a descending frequency. Figure 3B shows the calibration curve for bleeding events, with predicted probabilities mostly between 0.1 and 0.5.

## 4 Discussion

Our study demonstrates significant advancements in the field of post-PCI risk assessment for DAPT duration management. The transformer-based architecture coupled with auto-encoder and contrastive learning is adept at processing complex, large-scale datasets while capturing intricate patterns that are often missed by traditional machine learning models. This capability is crucial for understanding the complex dynamics of patient data post-PCI, allowing for a more accurate analysis of adverse endpoint risks. Moreover, our model performance is bolstered by the use of multiple loss functions that concurrently optimize different aspects of the prediction task. This multi-objective approach allows for a more balanced model that does not overly prioritize one predictive goal over another, thus maintaining a holistic view of the patient's risk profile.

By integrating the survival analysis mechanism, our model allows flexibility in risk assessment at any specific time point within the first year after PCI. This feature is particularly beneficial as it supports dynamic risk evaluation, adapting to the evolving clinical status of patients over time. Our model not only forecasts the probability of adverse events on the index date but also adjusts these predictions as new data becomes available, offering a robust tool for continuous patient monitoring.

The practicality of our model in clinical environments is facilitated by its requirement for a limited number of input features. These features, encompassing patients' demographics, clinical presentations, medical history, and concurrent medications, have been carefully chosen to balance comprehensiveness with easy data collection. This selectivity ensures that the model remains both user-friendly and efficient, minimizing the burden on healthcare providers while still capturing the necessary data to assess patient risk accurately. The ability of our model to provide timely and tailored risk assessments holds promise to enhance personalized treatment strategies. For instance, by identifying patients at higher risk of complications at any point within the year post-PCI, clinicians can tailor the duration of DAPT and other therapeutic interventions. This approach not only aims to optimize patient outcomes by preventing over or under-treatment but also contributes to the broader goals of personalized medicine, where treatment plans are customized to individual patient needs.

The strengths of our predictive model are multifaceted: (1) Excellent Predictive Accuracy. Our model consistently outperforms the state-of-art deep learning-based survival models, as demonstrated in Tables 2, 3, where most *p*-values are <0.05. Specifically, the model achieves C-index ranging from 0.88 to 0.80 for ischemic prediction and 0.82–0.77 for bleeding prediction across different prediction intervals. In comparison, the DAPT score achieves a C-index of 0.70 for ischemia and 0.68 for bleeding (7), indicating our model improves predictive accuracy by over 10%. (2) Enhanced Flexibility and Dynamic Prediction Capability: Unlike clinical trial-derived scores which often lack adaptability, our model is built on real-world data, enabling it to accommodate patients' evolving clinical status over time. Leveraging a deep survival infrastructure supported by the piecewise constant hazard loss, the model allows for dynamic risk evaluation at any specific time point within the prediction window following PCI. As new evidence emerges and clinical guidelines evolve, encompassing considerations for shorter DAPT duration, treatment de-escalation, and monotherapy (11, 36–39), there is an increasing need for more granular and personalized predictions. Our AI-driven approach, trained on large-scale data, captures the subtle nuances and delivers precise, individualized risk assessments, making it well-suited for addressing these emerging clinical demands.

The calibration curve serves as an essential diagnostic tool for understanding our predictive model's performance. Specifically, the calibration curve for predicting ischemic events (Figure 3A, red line) closely aligns with the ideal line (Figure 3A, blue dashed line) across much of the predicted probability spectrum, indicating that our model is well-calibrated for predicting ischemic events over a broad range of probabilities. Conversely, for bleeding event predictions, the calibration curve deviates from the ideal scenario at lower probabilities (approximately 0.1–0.2), where the red line lies below the ideal line (Figure 3B), suggesting that the model tends to underestimate the risk of bleeding events at these lower probability values. As the predicted probabilities increase to about 0.3, the calibration curve approaches the ideal line, indicating an improvement in predictive accuracy. However, at higher probabilities, the curve extends above the ideal line, implying that the model overestimates the risk of bleeding events at these higher probabilities. The accompanying histogram shows that the predicted probabilities for bleeding events are predominantly clustered within the 0.1–0.4 range. This clustering suggests a conservative prediction behavior by the model, likely cautious about assigning higher probabilities for bleeding events. This pattern could be attributed to an inherent class imbalance in our dataset, with the ratio of the event to the non-event being approximately 5.25 in our study cohort. To enhance model calibration and performance, especially in different probability bins, it is beneficial to address the class imbalance issue. Techniques such as the Synthetic Minority Over-sampling Technique (SMOTE) (40) or adjusting the dataset to a more balanced distribution could potentially refine the model's accuracy across the full range of predicted probabilities.

Despite several strengths, our study has some limitations. A primary challenge derives from the quality of the EHR data, which is inherently prone to the issues of incompleteness and missingness. Specifically, crucial information such as stent characteristics, the severity of bleeding, and stent thrombosis were not available from the structured EHR data alone. Future research would benefit from incorporating information extracted from clinical notes, to enhance the robustness of our model. Another limitation relates to the loss of follow-up information. Some patients returned for follow-up long after their initial visit, potentially due to the prescriptions being auto-filled for several months or because they received care at other hospitals. To manage this, we implemented a 6-month window threshold. If DAPT medications were prescribed within this timeframe, patients were assumed to have continued their DAPT regimen. This assumption requires further validation, either through additional patient data or confirmation by clinical expertise. Furthermore, our study primarily utilized ICD codes to define features and outcomes. Although these codes were carefully selected based on previous research and reviewed by our clinician collaborators, continuous verification of the reliability of these codes is necessary to strengthen the conclusions drawn from our findings. In addition, this study utilizes the multi-institution, diverse dataset from the OneFlorida^+^ Clinical Research Consortium, which encompasses approximately 16 million patients across the Southeast—including Florida, Georgia, and Alabama (27, 28)—serving as a robust and representative resource for model development, given its scale, diversity, and regional significance. While we believe this dataset currently satisfies the needs for developing and evaluating the proposed algorithm, we plan to pursue external validation using other high-quality datasets if available in the future, to further test the model's generalizability across broader populations.

For future efforts, first, we will leverage all medical codes, instead of the selected features, as another version of the input, seeking to further enhance the model performance. Our model is inherently designed to handle complex and high-dimensional data and is well-suited to learn from the intricacies presented in raw medical codes. Second, given the limitations of structured data, we plan to incorporate higher-quality, multimodal data, such as clinical notes and medical imaging, to augment input information. Clinical notes often contain rich, contextual information that can complement structured data, while medical imaging can provide crucial insights into a patient's condition that are not captured in traditional data formats. By integrating these diverse data sources, we aim to create a more comprehensive and accurate representation of patient profiles. Third, to effectively harness these multimodal data, we will upgrade our model to support multimodality learning (41). This approach will enable the model to simultaneously process and learn from different types of data, improving its predictive capabilities and robustness. Fourth, in addition to focusing on data and model improvements, we will align our research goals more closely with the evolving clinical needs. Beyond optimization of treatment durations, we also plan to explore dual-single antiplatelet succession strategies, which involve investigating the effectiveness of transitioning patients between different treatment regimens, potentially improving outcomes by tailoring strategies to individual needs and responses. By pursuing these future efforts, we hope to develop more powerful and versatile AI solutions that can provide valuable insights to clinicians, thus facilitating better patient care and outcomes. Fifth, the focus of this current paper is to develop and evaluate the AI model. To enhance its accessibility and maximize its utility to the wider community, we plan to create a user-friendly website, which allows for both manual input of the commonly available clinical variables and the upload of comprehensive patient records, to enable efficient and accurate risk calculations. Furthermore, to demonstrate the model's functionality and strengths—such as accommodating dynamic input and delivering flexible predictions—the model can be potentially embedded as an API into our EHR systems to evaluate its usability and real-world performance.

## 5 Conclusion

In this study, we introduce a new, effective method for predicting risks in patients receiving DAPT following PCI. Our transformer-based model, enhanced by contrastive learning, consistently outperforms existing deep survival models. This model enhances flexibility in prediction, substantially aiding the clinical decision-making process. Moving forward, it is essential to prioritize the enhancement of data quality and modality to boost both the accuracy and clinical applicability of AI models. Our methodology provides a robust foundation for developing more precise and personalized antiplatelet therapy strategies, offering significant potential to improve patient outcomes and save lives.

## Data Availability

The dataset for this study is not publicly available due to privacy and ethical restrictions. Data supporting the findings of this study can be accessed through the OneFlorida^+^ clinical research network. A data request can be submitted via https://onefloridaconsortium.org/, and a data use agreement will be needed.
