# Supplementary material for: Contrastive learning with transformer for adverse endpoint prediction in patients on DAPT post-coronary stent implantation
Source: Front Cardiovasc Med. 2025 Jan 13;11:1460354. doi: 10.3389/fcvm.2024.1460354 (PMC11769931; doi:10.3389/fcvm.2024.1460354)
Supplement: Supplementary file 1 [file Table1.docx]

**Supplementary File**

Table S1 Hyperparameters of the Proposed Model for Ischemic and Bleeding Event Predictions

| **Hyperparameters** | **Description of Hyperparameters** | **Values of Hyperparameters** | |
| --- | --- | --- | --- |
|  |  | **Ischemic Event Prediction** | **Bleeding Event Prediction** |
| optimizer_name | Name of optimizer | Adam | BERTAdam |
| balanced | Flag for whether balancing the ratio of patients with events vs. without events | False | False |
| lr | Learning rate | 0.002 | 0.002 |
| batch_size | Batch size | 256 | 64 |
| embedding_size | Embedding size | 128 | 112 |
| num_hidden_layers | Number of hidden layers | 2 | 1 |
| hidden_size | Latent layer size of auto-encoder | 40 | 8 |
| intermediate_size | Intermediate layer size in transformer | 40 | 8 |
| focal | Flag for whether using focal loss in BCE for handling imbalanced data classes | False | True |
| MSE_weight | Weight of MSE in auto-encoder | 0.300 | 0.9 |
| Triplet_weight | Weight of contractive learning loss function | 0.137 | 0.2 |
| BCE_weight | Weight of binary cross entropy loss function | 0.14 | 0.2 |
| hazard_weight | Weight of piecewise constant hazard loss function | 0.95 | 0.89 |
| num_durations | Number of durations | 12 | 6 |
| num_sub | Number of sub-intervals within each duration | 7 | 10 |
| c_td_version | Version of the time dependent C-index | antolini | antolini |
| num_attention_head | Number of attention head of transformer | 2 | 2 |
| attention_probs_dropout_prob | Dropout probability in attention layer | 0.1 | 0.1 |

Abbreviations: BCE, binary cross entropy; MSE, mean squared error
